# Retraction: Microchip-based structure determination of low-molecular weight proteins using cryo-electron microscopy

**DOI:** 10.1039/d4nr90151g

**Published:** 2024-08-12

**Authors:** Michael A. Casasanta, G. M. Jonaid, Liam Kaylor, William Y. Luqiu, Maria J. Solares, Mariah L. Schroen, William J. Dearnaley, Jarad Wilson, Madeline J. Dukes, Deborah F. Kelly

**Affiliations:** a Department of Biomedical Engineering, Pennsylvania State University University Park PA 16802 USA Debkelly@psu.edu; b Materials Research Institute, Pennsylvania State University University Park PA 16802 USA; c Bioinformatics and Genomics Graduate Program, Huck Institutes of the Life Sciences, Pennsylvania State University University Park PA 16802 USA; d Molecular, Cellular, and Integrative Biosciences Graduate Program, Huck Institutes of the Life Sciences, Pennsylvania State University University Park PA 16802 USA; e Department of Electrical and Computer Engineering, Duke University Durham NC 27708 USA; f RayBiotech Life Peachtree Corners GA 30092 USA; g Applications Science, Protochips, Inc Morrisville NC 27560 USA

## Abstract

Retraction of ‘Microchip-based structure determination of low-molecular weight proteins using cryo-electron microscopy’ by Michael A. Casasanta *et al.*, *Nanoscale*, 2021, **13**, 7285–7293, https://doi.org/10.1039/D1NR00388G.

The Royal Society of Chemistry hereby wholly retract this *Nanoscale* paper due to concerns about the reliability of the electron microscopy data. We were alerted by a reader about possible concerns with the electron microscopy data. We have investigated the data with the support of independent experts.

The pixel values cited in the article are not consistent with the deposited EMDB entries EMD-22982 and EMD-24147.

In the opinion of experts the ultra-low dose conditions used to collect the cryo-EM images of frozen-hydrated N-protein particles, (<5 electrons per Å^2^) at 200 kV (Fig. 2A), mean the alignment of images was not possible, as described by Poisson statistics.

More high-frequency noise is observed in the full maps deposited as EMDB entries EMD-22982 and EMD-24147 compared to the half maps supplied by the authors for expert assessment. Whereas it would be expected that noise would reduce when more particles are averaged together.

The stated pixel size and volume dimensions also differ between the half maps and full maps.

The protein data bank (PDB) model (supplied by the authors for expert assessment) contained B-factor values and ATOM lines for explicit hydrogen atoms that could not be reproduced in PHYRE2 runs on the N-protein sequence.

On independent analysis, a map:model Fourier shell correlation (FSC) curve produced using the author-supplied PDB file and the final map in EMD-24147, with a threshold of 0.5 for comparison, returned a judged resolution of 8.6 Å. This is not consistent with the results reported in the manuscript.

Independent experts were consulted who were not satisfied with the explanation provided by the authors, therefore this article is being retracted to protect the integrity and accuracy of the scientific record.

Deborah F. Kelly opposes the retraction and maintains the validity of the published results.

Michael A. Casasanta, G. M. Jonaid, Liam Kaylor, William Y. Luqiu, Mariah L. Schroen, and William J. Dearnaley were contacted but did not respond.

Madeline J. Dukes has responded to indicate that she does not oppose nor endorse the retraction notice.

Jarad Wilson has not indicated that he opposes or endorses the retraction notice.

This retraction supersedes the information provided in the Expression of Concern related to this article.

Signed: Maria J. Solares

Date: 5/8/2024

Retraction endorsed by Heather Montgomery, Managing Editor, *Nanoscale*

